# Prognostic value of tumor-infiltrating lymphocytes, tumor associated neutrophils and metabolic checkpoint molecules on survival of patients with metastatic pancreatic ductal adenocarcinoma

**DOI:** 10.1016/j.sipas.2025.100320

**Published:** 2025-11-30

**Authors:** Tao Zhang, Rainer C. Miksch, Ughur Aghamaliyev, Maximilian Weniger, Michael Günther, Steffen Ormanns, Fatma Parsa, Jan G. D’Haese, Alexandr V. Bazhin, Matthias Ilmer, Bernhard W. Renz, Jens Werner

**Affiliations:** a Department of General, Visceral and Transplantation Surgery, LMU University hospital, Munich, Germany; b Department of Hepatobiliary Surgery, Chinese PLA Air Force Medical Center, Beijing 100142, China; c Department of Pathology, Ludwig-Maximilians-University Munich, Marchioninistr. 15, 81377 Munich, Germany; d German Cancer Consortium (DKTK), Partner Site Munich, Germany; e Bavarian Cancer Research Center (BZKF), 91054 Erlangen, Germany

**Keywords:** Tumor-infiltrating lymphocytes, Metabolic checkpoint molecules, pancreatic ductal adenocarcinoma, Tumor microenvironment, Tumor stroma

## Abstract

**Objectives:**

This study aimed to investigate the tumor microenvironment (TME) of metastatic PDAC, focusing on tumor-infiltrating leukocytes (TILs) and metabolic checkpoint molecules (MCMs).

**Background:**

The role of TME in primary and metastatic PDAC is not well understood. Furthermore, the role of energy metabolism in metastatic PDAC is unclear. Therefore, this study aimed to explore the TME in primary tumors and metastases of PDAC, and its prognostic role.

**Materials and Methods:**

We included 26 cases of metastatic PDAC in this study. We performed immunohistochemistry for TILs and MCMs (HIF-1α, GLUT1, and PDHK1) in primary and corresponding metastatic tumor tissues. We quantified stromal TILs and MCMs using a tumor immune stroma (QTiS) algorithm and correlated the data with clinical outcome.

**Results:**

We found that CD3^+^, CD8^+^, and CD20^+^ TILs were increased in primary tumors compared to metastatic ones. Kaplan-Meier plots revealed that high infiltration of CD20^+^ and its combinations in primary tumors correlated with better OS in metastatic PDAC patients. We also found that high infiltration of CD8^+^ TILs in metastatic tumors correlated with better OS, as did the low density of GLUT1 in both PDAC primary and metastatic tumors. A multivariate Cox regression analysis revealed that CD8^+^ TILs in metastatic tumors and GLUT1 in PDAC primary and metastatic tumors were independent predictors of survival.

**Conclusion:**

Distribution of TILs in the TMEs of primary and metastases of metastatic PDAC is different. Our results suggest that TILs (CD8^+^) and MCMs (GLUT1) in tumor stromal areas can predict OS of patients with metastatic PDAC.

## Introduction

Pancreatic ductal adenocarcinoma (PDAC) remains a formidable challenge for clinicians due to its high mortality rate worldwide [1]. Early diagnosis is essential for successful treatment as most cases present in advanced tumor stages, leaving surgery as the only curative option [2]. Unfortunately, PDAC’s incidence is expected to rise [2]. The tumor microenvironment (TME) of PDAC is stromal-dominated and capable of evading immune responses and immune-modulated therapies [3]. This heterogeneous cellular environment acts in concert to reduce tumor immunity [4].

Energy metabolism within the TME is a significant driver of cancer progression and plays a critical role in the growth, invasion, and metastasis of PDAC [5,6]. T and B lymphocytes are essential components of the TME and have been demonstrated to predict prognosis in breast, lung, and colorectal cancer [[7], [8], [9]]. Preliminary studies have shown that Tumor-Infiltrating Leukocytes (TILs) correlate with PDAC’s prognosis [10,11]. It also has been demonstrated, that Quantification of the tumor immune stroma (QTiS) predicts survival in PDAC patients [10]. However, data on the composition of the TME in primary tumors and its relationship to the corresponding metastatic lesion in PDAC remain limited. PDAC has the ability to evade the immune system, making immune-based therapies less effective than in other solid tumors [12]. This immune evasion relies on mechanisms such as stromal fibrosis within a hypoxic TME, heavily influenced by metabolic alterations [13]. In this regard Glucose metabolism, in particular, is fundamental to PDAC progression [14]. Glucose metabolism plays a central role in regulating immune cell activation, proliferation, and function. Within the TME, competition for glucose among tumor cells, immune cells, stromal cells, and lymphocytes contributes to T-cell dysfunction and immune evasion [15,16]. Reducing this metabolic competition between tumor cells and the immune system has emerged as a promising therapeutic approach [17]. The Warburg effect, which highlights cancer cells’ preference for glycolysis, contributes to the altered microenvironment and increased invasiveness seen in PDAC [[18], [19], [20]]. Specifically, overexpression of glucose transporter 1 (GLUT1) and pyruvate dehydrogenase kinase 1 (PDHK1) in hypoxic conditions promotes anaerobic glycolysis, leading to lactate production that acidifies the TME and facilitates tumor progression, drug resistance, metastasis, and immune escape [21].

PDAC is marked by a desmoplastic stroma and low vessel density, which further reinforces its resistance to therapy [22]. Currently, there are numerous conflicting reports discussing the significance of tumor-associated neutrophils (TANs) and tumor-infiltrating lymphocytes (TILs) in PDAC. In this study, we aimed to examine the prognostic value of TILs CD3^+^, CD8^+^, CD20^+^ TILs and tumor-associated neutrophils (CD66b^+^) TANs on PDAC patients’ survival [10,23]. Furthermore, we also investigated the prognostic value of metabolic checkpoint molecules MCMs (HIF-1α, GLUT1, and PDHK1).

## Material and methods

### Patients and clinical data

In the current study, all tissue specimens used for staining were extracted from the archive of the Institute of Pathology of Ludwig-Maximilians-University, Munich. The tumor samples were anonymized, as mandated by the HTCR Foundation and the Declaration of Helsinki. Before the initiation of the study, we obtained approval from the institutional ethics committee of LMU in Munich (Project No.19–257).

We included tumor samples from 26 patients who underwent resection at our institution between November 2001 and October 2018. The clinical characteristics of these patients, including sex, age, synchronous or metachronous metastasis relative to initial pancreatic cancer, sites of metastasis (i.e., liver, lung, or peritoneum), TNM stage, and preoperative laboratory values (Suppl. Table 2) were prospectively collected and recorded in an institutional database. Additionally, platelet counts of all patients were examined by the laboratory medicine department and included in our database.

### Demographical data

As mentioned above, twenty-six (26) metastatic PDAC patients were included in this study, 20 of whom with synchronous metastases, and 6 with metachronous metastases. out of these, 18 had liver metastases, 6 peritoneal metastases, and 2 lung metastases. Patient’s age ranged from 44 to 78 years, with a median age of 63.8 years. 57.7 % of the patients were female. Seven pylorus resecting pancreaticoduodenectomies (PrPD), six pylorus-preserving pancreaticoduodenectomies (PPPD), twelve left pancreatectomies, and one total N

Npancreatectomy were performed (Supplementary Table 1).

The median disease-free survival (DFS) was 7.0 months (standard deviation (SD) ± 25.262), and the median overall survival (OS) was 11.0 months (SD ± 45.343). Twenty (20) patients underwent simultaneous resection of primary and metastatic disease. Six (6) patients developed distant tumor metastases during follow-up, including one case with two times pulmonary metastases, and all six (6) patients underwent resection of metastatic tumors after confirmation of metastases. Eighteen patients (69.2 %) finished all cycles of adjuvant chemotherapy with gemcitabine, while the remaining eight patients (30.8 %) discontinued chemotherapy due to intolerance or death.

### Material

All 4 µm thick sections used for staining were obtained from the Institute of Pathology of Ludwig-Maximilians-University, Munich. Primary antibodies including anti-CD3 (clone SP7, SpringBio, Pleasanton, CA, USA), anti-CD8 (clone C8/144B, Dako, Glostrup, Denmark), anti-CD20 (clone L26, Dako, Glostrup, Denmark), anti-CD66b (ab197678, Abcam, UK), anti-GLUT-1 (clone SPM498, Abcam, Cambridge, UK), anti-HIF-1α antibody (clone 54/HIF-1α, BD Biosciences, San Jose CA, USA), and anti-PDHK1 (Ab110025, Abcam, UK) were procured from reputable companies in the USA, UK, and Denmark. We obtained secondary antibodies (horse anti-mouse IgG (BA-2000) and horse anti-rabbit IgG (BA-1100)) from VECTOR Laboratories (Burlingame, CA, USA), along with the Avidin/Biotin Blocking Kit (SP-2001) and UltraView Universal DAB Detection Kit, the alkaline phosphatase enzyme detection system (ABC-AP) (AK-5000), and the ImmPACT Vector Red Alkaline Phosphatase Substrate Kit (SK-5105).

### Analysis of immunohistochemistry

Following the established methodology [10,24], stromal regions displaying the highest concentration of CD3+, CD8+, CD20+, or CD66b+ infiltration/expression were identified as hot spots, indicating the accumulation of specific stained markers. Furthermore, single stained markers were also identified, as well as the absence of stained markers. Where feasible, three hot spots per antigen were assessed, and median values calculated (Supplementary Table 1). A similar hot spot selection and quantification approach was employed for GLUT1, HIF-1α, and PDHK1. Quantification of stained markers was carried out in three steps using ImageJ software (Version 1.51 h, National Institutes of Health, Bethesda, MD, USA). First, the original jpeg-format images were converted to 32-bit, and the subjective staining threshold was set using the ImageJ Standard tool to accurately visualize the contour of the target marker. The larger particles were subsequently separated using the ImageJ software's watershed function. Finally, the "analyze particles" function was utilized to automatically count these particles, completing the quantification of the target marker.

### Statistical analysis

The R packages (version 3.6.3) and SPSS (Version 26.0, IBM, USA) were employed for statistical analysis. A P-value of <0.050 was considered statistically significant, and univariate analysis results with P-values <0.050 were subjected to multivariate analysis. Kaplan-Meier survival analyses were conducted to estimate overall and disease-free survival rates. Additionally, univariate and multivariate Cox regression analyses were used to evaluate the dependent variables of overall survival and disease-free survival.

The immunohistochemistry results of seven stained markers were classified into high and low groups based on median values. Median values were calculated as the mean values for each antibody and each patient, followed by identifying the median from the 26 different mean values.

Statistical analysis was performed on high vs. low infiltration/expression for TILs and MCPs. For two markers analyzed together, groups were formed by separating high infiltration in both markers from the rest. For three markers analyzed together, groups were formed by separating the high group in all markers, the low group in all markers, and two high of three markers defined as the high group. Similarly, for four markers analyzed together, groups were formed by separating the high group in all markers, the low group in all markers, and three high of four markers defined as the high group. Accordingly, Kaplan-Meier analyses were performed for TILs and MCMs and their combinations. Spearman correlations were used to calculate the correlation of all the variables (including seven IHC staining markers and laboratory values) in the study. Furthermore, a nomogram model was constructed based on the multivariable Cox regression to validate the predictive value of TILs and MCMs for metastatic PDAC.

## Results

### Expression frequency of TILs and MCMs in primary tumors of metastatic PDAC patients correlates with survival

Immunohistochemistry (IHC) staining revealed positive labeling of TILs and MCMs (Supplementary Figure 1) in the stroma of primary tumors and metastatic lesions. The stromal areas with the highest infiltration/expression of CD3^+^, CD8^+^, CD20^+^, CD66b^+^, GLUT1, HIF-1α, and PDHK1 were defined as hot spots. The quantitative data of these seven markers in primary and metastasis are provided in Supplementary Table 1.

**Fig. 1:**
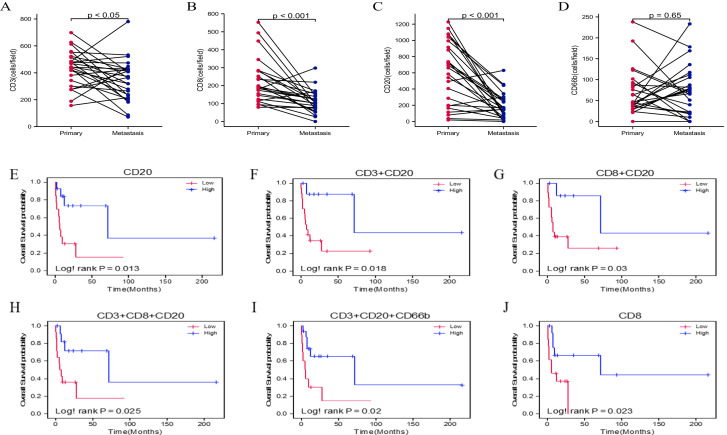
Correlation of expression frequency of TILs and MCMs with survival in primary tumors of metastatic PDAC patients. CD3+(A), CD8+(B), and CD20+ (C) were significantly increased in primary compared to metastatic tumors in metastatic PDAC. Infiltration of CD66b+TILs (D) did not reveal any statistical difference between primary and metastatic tumors. Overall survival for high (blue) vs. low (red) infiltration of CD20+ (E) and CD8+ (J) tumor-infiltrating leukocytes (TILs). After quantification of the stained cells, the median was used to distinguish high from low infiltration. High infiltration of CD20+ TILs from primary tumors and high infiltration of CD8+ from metastatic tumors of metastatic PDAC correlated significantly with improved OS. Survival graphs of OS in relation to different combinations of TILs: Groups were formed by separating high infiltration in both cell types (blue color, e.g., high CD20+ and high CD8+) from the rest (red color, e.g., high/low CD20+/CD8+ or low CD20+ and low CD8+), where two immune cells were analyzed together (F-G). Groups were formed by separating the high group in all cell types, the low group in all cell types, and two high of three cell types defined as the high group, where three immune cells were analyzed together (H-I). High infiltration of CD3++CD20+, CD8++CD20+, CD3++CD8++CD20+, and CD3++CD20++CD66b+ tumor-infiltrating leukocytes correlated significantly with better OS.

The quantitative analysis of CD3^+^, CD8^+^, CD20^+^, and CD66b^+^ TILs from 26 metastasized PDACs was grouped into primary and metastatic categories for pairwise comparison. Results indicated that CD3^+^(*p* < 0.05), CD8^+^(*p* < 0.001), and CD20^+^(*p* < 0.001) TIL infiltration levels were significantly higher in (metastasized) primary tumors compared to metastatic sites. In contrast, CD66b^+^TIL infiltration levels were not different (*p* = 0.65) (Fig. 1).

For survival analysis, infiltration levels of CD3+, CD8+, CD20+, and CD66b+ TILs were categorized into high and low groups based on median values (Supplementary Table 1). Kaplan-Meier plots showed that high CD20^+^infiltration in primary tumors was significantly associated with improved OS (Log-rank *P* = 0.013) (Fig. 1E). Further analysis of combinations of leukocyte infiltrations revealed that high levels of CD3+CD20+ (Log-rank *p* = 0.018), CD8+CD20+ (Log-rank *p* = 0.03), CD3+CD8+CD20+ (Log-rank *p* = 0.025), and CD3+CD20+CD66b+ (Log-rank *p* = 0.02) in primary tumors were significantly linked to improved OS in metastatic PDAC (Fig. 1F-I). Additionally, high CD8+ infiltration in metastatic tumors was also significantly associated with better OS (Log-rank *p* = 0.023) (Fig. 1J). However, no significant association was found between CD3+ or CD66b+ TIL infiltration and OS, nor were there any significant differences in TIL infiltration impacting disease-free survival (DFS) (Supplementary Figure 1B).

### Expression of GLUT1 correlates with overall survival of metastatic PDAC patients

**Fig. 2:**
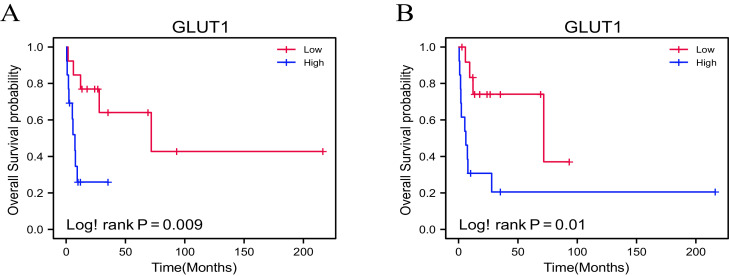
Overall survival for high (blue) vs. low (red) scores of GLUT1. After quantification of the stained molecules, the median was used to distinguish high from low infiltration. Low scores of GLUT1 from both primary tumors (A) and metastatic tumors (B) correlated significantly with improved OS.

The quantitative analysis of GLUT1, HIF-1α, and PDHK1 in primary and metastatic tumors across 26 metastatic PDAC cases was grouped into primary and metastatic categories for pairwise comparison. No statistically significant differences in MCM expression were observed between primary and metastatic tumors (Supplementary Figure 2). For survival analysis, GLUT1, HIF-1α, and PDHK1 expression levels were categorized into high and low expression levels based on median values (Supplementary Table 1). Kaplan-Meier plots indicated that low GLUT1 expression in both primary (Log-rank *p* = 0.009) and metastatic tumors (Log-rank *p* = 0.01) was significantly associated with improved OS (Fig. 2). However, no significant association with OS or DFS was found for HIF-1α, PDHK1, or any combination of the three MCM markers (Supplementary Figure 4).

### Expression of TILs and MCMs correlates with laboratory values of PDAC patients

In the primary tumor of metastatic PDAC, CD3 infiltration was positively correlated with CD20 (*p* < 0.01) and preoperative blood amylase (*p* < 0.05), but negatively correlated with PDHK1 in the metastasis (*p* < 0.05). CD3 infiltration in metastases was positively correlated with CD8 (*p* < 0.01), CD20 M (*p* < 0.05), and CD66b (*p* < 0.05). CD8 P showed a positive correlation with CD20 (*p* < 0.05), while CD8 was positively correlated with both CD20 (*p* < 0.05) and CD66b (*p* < 0.05). Additionally, CD20 correlated with CD66b (*p* < 0.05), CD66b, preoperative platelet count, and CRP levels in the primary tumor (*p* < 0.01), as well as with Crea, GGT, and INR (*p* < 0.05).

**Fig. 3:**
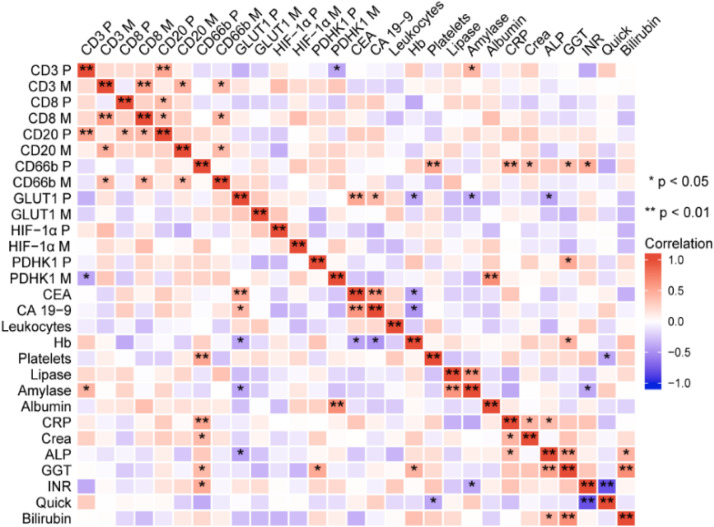
Correlation heatmap with statistical significance represented by the pentagram. Heat map indicates clustering and positive correlation of TILs and MCMs with serum parameters of PDAC patients including CEA, CA19–9, Amylase, Lipase, Hb, Leukocytes, CRP, Creatinine, Bilirubin, GGT. Colors indicate Pearson r from 1 (red) to −1 (blue); ⭑⭑indicates a highly significant correlation (*p* < 0.01), ⭑indicates a significant correlation (*p* < 0.05). (Abbreviations: CEA: Carcinoma Embryonic Antigen; CA19–9: Carbohydrate antigen 19–9; Hb: Hemoglobin; CRP: C-reactive protein; Crea: Creatinine; ALP: alkaline phosphatase; GGT: Gamma-glutamyl transpeptidase; INR: international normalized ratio. Note: **P** stands for Primary tumor, **M** stands for metastasis tumor).

Interestingly, GLUT1 expression in the primary tumor correlated positively with preoperative serum CEA (*p* < 0.01) and CA-19–9 (*p* < 0.05), but negatively with preoperative blood Hb, amylase, and ALP (*p* < 0.05). PDHK1 P was positively correlated with preoperative blood GGT (*p* < 0.05), while PDHK1 M showed a strong positive correlation with preoperative blood albumin (*p* < 0.01) (Fig. 3).

Preoperative blood CEA levels were strongly positively correlated with CA-19–9 (*p* < 0.01). Hb in preoperative blood also correlated positively with CEA, CA-199, and GGT (*p* < 0.05). Platelets were negatively correlated with Quick (*p* < 0.05), and amylase was negatively correlated with INR (*p* < 0.05) but strongly positively correlated with lipase (*p* < 0.01). CRP was positively correlated with Crea and ALP (*p* < 0.05). ALP was strongly positively correlated with GGT (*p* < 0.01) and positively correlated with bilirubin (*p* < 0.05). GGT also had a strong positive correlation with bilirubin (*p* < 0.01), and INR was strongly negatively correlated with Quick (*p* < 0.01) in this study (Fig. 3).

### CD8+ cells and GLUT1 are independent variables for predicting OS of metastatic PDAC patients

Univariate Cox regression analysis was performed on seven markers, laboratory values, and clinical parameters, with OS and DFS as the dependent variables (due to the potential statistical consequences of double-use, combination groups of different immune cell types and metabolic checkpoint molecules were excluded) (Supplemental Table 1). Seven IHC markers and laboratory values were categorized into high and low infiltration/expression groups, using median values as the cutoff, for Cox regression analysis. In the univariate Cox regression analysis, CD20^+^P (HR: 0.250, *p* = 0.021,95 %CI: 0.080–0.810), CD8^+^M (HR: 0.270, *p* = 0.033, 95 %CI: 0.080–0.900), GLUT1 P (HR: 4.687, *p* = 0.015, 95 %CI: 1.347–16.306), GLUT1 M (HR: 4.137, *p* = 0.018, 95 %CI: 1.281–13.357), and platelets (HR: 4.038, *p* = 0.022, 95 %CI: 1.225–13.313) were statistically significant on OS. Supplemental Table 1 shows that high infiltration of CD20^+^TILs (*p* = 0.021) in primary tumors of metastatic PDAC and high infiltration of CD8^+^ TILs (*p* = 0.033) in metastatic lesions correlated with improved OS; low density of GLUT1, both in primary tumors and metastasis correlated with improved OS; low levels of platelets correlated with improved OS. However, there was no variable associated with DFS.

Multivariate analysis was performed using variables with p-values < 0.050 in univariate analysis, and statistical significance was considered for p-values < 0.050. In the case of multivariate Cox regression analysis (Table 1), we found that CD8^+^ *M* (HR: 0.196, *p* = 0.032, 95 %CI: 0.044–0.872), GLUT1 P (HR: 5.816, *p* = 0.049, 95 %CI: 1.006–33.624), and GLUT1 M (HR: 5.056, *p* = 0.022, 95 %CI: 1.258–20.324) were statistically significant predictor for OS. High infiltration of CD8^+^ TILs in metastatic lesions and low expression of GLUT1 in both primary and metastatic lesions correlated well with improved OS. More importantly, CD8^+^ *M*, GLUT1 P, and GLUT1 M independently predicted overall survival (OS) of metastatic PDAC.

**Table 1:** Multivariate cox regression analysis. P stands for Primary tumor, M stands for metastatic tumor, significant p-values are displayed in bold (n = 26).

Variables	Overall survival			
	HR	95% CI		p-value
CD20 **P**	0.272	0.066	1.124	0.072
CD8 **M**	0.196	0.044	0.872	**0.032**
GLUT1 **P**	5.816	1.006	33.624	**0.049**
GLUT1 **M**	5.056	1.258	20.324	**0.022**
Platelets	3.706	0.769	17.851	0.102

**Fig. 4:**
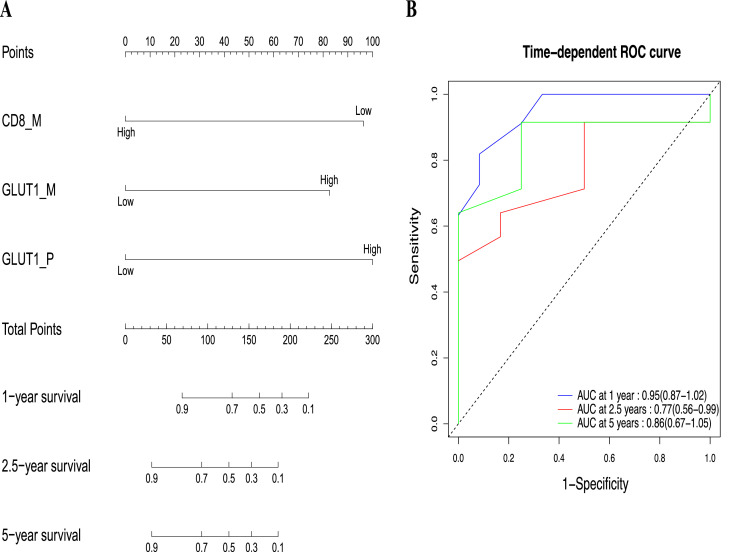
Nomogram construction and validation. (A) A nomogram with CD8 M, GLUT1 P, and GLUT1M predicting the overall survival probability of 1-, 2.5-, and 5-years in metastatic PDAC. (B) Time-dependent ROC analyses of CD8 M, GLUT1 P, and GLUT1M predict 1-, 2.5-, and 5-year overall survival. P stands for Primary tumor of PDAC; M stands for metastatic tumor of PDAC.

Based on the multivariable Cox regression, CD8 M, GLUT1 P, and GLUT1M are independent predictors of OS in metastatic PDAC patients. The nomogram model and ROC curves (Fig. 4), predict time-dependent the accuracy of the three markers on OS. The AUC values at 1, 2.5, and 5 years were 0.95 (95 %CI: 0.87–1.02), 0.77 (95 %CI: 0.56–0.99), and 0.86 (95 %CI: 0.67–1.05), respectively.

## Discussion

PDAC remains a highly lethal disease with a dismal prognosis, and its incidence and associated health burden continue to rise annually [2]. Although various treatment approaches exist, only around 25 % of PDAC patients are eligible for curative resection [25]. The TME of PDAC is composed of immune cells, cytokines, stromal fibroblasts, and ECM, creating a complex environment that supports pancreatic cancer growth, metastasis, and resistance to therapy [26]. This intricate TME may contribute to the limited success of multiple treatment strategies, including chemotherapy, radiotherapy, and immunotherapy.

Diagnosing and treating PDAC continues to present challenges in clinical practice. To our knowledge, this study involves the largest cohort comparing primary and corresponding metastatic tumors. We analyzed infiltration levels of CD3+, CD8+, CD20+, and CD66b+ TILs between primary and corresponding metastatic lesions. Our findings show that lymphocyte infiltration (CD3+, CD8+, and CD20+ TILs) was higher in primary tumors of metastasized PDAC compared to the metastatic lesion. Our findings on primary tumors align with previous research in other tumor types, suggesting that immune evasion contributes to tumor progression [27]. Prior studies have shown that CD3+ or CD8+ cells possess predictive value in cancers such as hepatocellular carcinoma, colorectal cancer, and breast cancer [8,9,28]. Additionally, a TME densely infiltrated by CD3+ or CD8+ *T* lymphocytes is considered a positive prognostic marker in PDAC [10]. High levels of CD3+ *T* lymphocyte infiltration correlate with improved overall survival in PDAC. CD8+ cytotoxic T lymphocytes, which secrete perforin and granzyme and express Fas ligands, play a critical antitumor role [29]. Multiple studies have highlighted the association between elevated CD8+ infiltration and extended survival in PDAC. Tumor-infiltrating B lymphocytes may directly kill tumor cells via an antibody‐independent approach and promote cell‐mediated immunity [30]. Regulatory T cells may inhibit B cell activation, proliferation, and antibody production [31]. Our previous data demonstrated the favorable effect of high infiltration of CD20+ *B*-lymphocyte on survival in PDAC [10]. The present study is the first to elucidate the function of stromal tumor-infiltrating leukocytes in metastatic PDAC. High levels of infiltration of CD20+ *B* cells in primary tumors were associated with improved OS; and high levels of CD8+ *T* lymphocyte infiltrations in metastatic tumors correlated with better OS. Notably, CD8+ *T* lymphocytes in metastatic tumors of PDAC were an independent predictor for OS.

Importantly, TIL therapy has already produced encouraging signals in early-phase studies in other solid tumors. For example, in a phase II study in metastatic melanoma evaluating TILs together with low-dose subcutaneous IL-2, a proportion of patients achieved partial responses, while others experienced disease stabilization, indicating clinically relevant activity [32]. In PDAC, TIL-based approaches have now also entered early clinical testing (phase I/II). However, results from these studies have not yet been reported, and the clinical efficacy of TIL therapy in PDAC therefore remains to be determined [33].

TME is a complex and heterogeneous ecosystem formed by cancer cells, immune and stromal cells through constant interactions [34]. A previous study has revealed that the TME is rich in components that influence tumor progression [4]. Glucose metabolism plays an important role in immune escape, metastasis, and drug resistance by participating in energy metabolism [34]. Hypoxia is a hallmark of TME in solid tumors due to the imbalance between increased oxygen utilization and inadequate oxygen supply [35]. Furthermore, hypoxia is a complex factor in tumor invasion and metastasis in the tumor microenvironment [36].

We quantified GLUT1, HIF-1α, and PDHK1 by the QTiS algorithm and found that low expression of GLUT1 in both primary and metastatic tumors correlated with improved OS. Notably, GLUT1 in primary and metastatic tumors, as well as CD8+ in metastatic tumors, were independent predictors for OS. Our results are in line with previous studies describing the prognostic value of HIF-1α and GLUT1 in patients with PDAC and PDHK1 in non-small cell lung cancer [[37], [38], [39]]. Also, in intrahepatic cholangiocarcinoma (iCCA), GLUT1 upregulation was shown to correlate with poor prognosis. Moreover, inhibition of GLUT1 reduced the proliferation, motility, and invasiveness of iCCA cells in vitro and in vivo [40]

Furthermore, the present study investigated the correlation between IHC markers and laboratory data. Several studies have revealed a positive correlation between CD20+ *B* lymphocytes and CD8+ *T* lymphocytes, such as in colorectal cancer and breast cancer [41,42]. Our study on metastatic PDAC also showed the correlation between CD20+ *B* cells and CD8+ *T* cells. In non-small cell lung cancer, a relationship between inflammatory markers CRP and CD66b+ TANs has been demonstrated [43], and the same result was found in our present work.

The TME plays a crucial role in tumor growth, invasion, and metastasis. Tumor and stromal cells intensely compete for survival, particularly in terms of nutrient consumption. This nutrient metabolism produces various byproducts, including lactate, which is linked to promoting tumor progression, drug resistance, metastasis, and immune evasion. Consequently, research on targeting GLUT1 and PDHK1 is ongoing. This study contributes to understanding the infiltration of TILs, TANs and MCMs in both primary and metastatic lesions of metastatic PDAC.

In Conclusion, the present findings demonstrate that survival of patients with metastatic pancreatic ductal adenocarcinoma (PDAC) is influenced by immune cells and metabolic checkpoint molecules. Infiltration rates of peritumoral CD8+ tumor-infiltrating leukocytes (TILs) in metastatic tumors and CD20+ TILs in primary tumors have been shown to correlate with improved overall survival (OS) in metastatic PDAC. Importantly, both CD8+ TILs in metastatic tumors and GLUT1 expression in primary and metastatic lesions have been identified as independent factors predicting OS in patients with metastatic PDAC. These findings emphasize the need for subtype classification of the tumor microenvironment (TME) in metastatic PDAC to accurately predict survival.

## Funding

This study was supported by a grant from the Förderprogramm für Forschung und Lehre (FöFoLe) der Medizinischen Fakultät der LMU München to R.C.M. (Reg.-Nr. 1037).

## CRediT authorship contribution statement

**Tao Zhang:** Writing – original draft, Methodology, Investigation, Formal analysis, Data curation, Conceptualization. **Rainer C. Miksch:** Writing – review & editing, Writing – original draft, Investigation, Funding acquisition, Data curation, Conceptualization. **Ughur Aghamaliyev:** Writing – review & editing, Data curation, Conceptualization. **Maximilian Weniger:** Writing – review & editing, Project administration, Methodology, Investigation, Conceptualization. **Michael Günther:** Writing – review & editing, Project administration, Methodology, Investigation, Conceptualization. **Steffen Ormanns:** Writing – review & editing, Methodology, Investigation, Conceptualization. **Fatma Parsa:** Writing – review & editing, Formal analysis, Conceptualization. **Jan G. D’Haese:** Writing – review & editing, Validation, Supervision, Project administration, Methodology, Conceptualization. **Alexandr V. Bazhin:** Writing – review & editing, Validation, Supervision, Resources, Project administration, Investigation, Conceptualization. **Matthias Ilmer:** Writing – review & editing, Writing – original draft, Resources, Project administration, Conceptualization. **Bernhard W. Renz:** Writing – review & editing, Validation, Supervision, Resources, Methodology, Data curation, Conceptualization. **Jens Werner:** Writing – review & editing, Validation, Supervision, Software, Resources, Investigation, Conceptualization.

## Declaration of competing interest

The authors declare the following financial interests/personal relationships which may be considered as potential competing interests:

Rainer C. Miksch reports financial support was provided by Förderprogramm für Forschung und Lehre (FöFoLe) der Medizinischen Fakultät der LMU München. The other authors declare that they have no known competing financial interests or personal relationships that could have appeared to influence the work reported in this paper.

## Appendix Supplementary materials

Image, application 1
