# Supplementary material for: Prognostic value of tumor-infiltrating lymphocytes, tumor associated neutrophils and metabolic checkpoint molecules on survival of patients with metastatic pancreatic ductal adenocarcinoma
Source: Surg Pract Sci. 2025 Nov 30;23:100320. doi: 10.1016/j.sipas.2025.100320 (PMC12721311; doi:10.1016/j.sipas.2025.100320)
Supplement: Supplementary file 1 [file mmc1.docx]

**Supplementary materials**

1. **Demographics of the study population.**

Abbreviations: SD: standard deviation; PrPD: pylorus resections pancreaticoduodenectomy; PPPD: pylorus-preserving pancreaticoduodenectomy; LP.:  left pancreatectomy; PC: pancreatic cancer; AFP: serum alpha‐fetoprotein; ALT: alanine transaminase; AST: aspartate transaminase; CRP: C‐reactive protein; INR: international normalized ratio of prothrombin time; GGT: Gamma-glutamyl transpeptidase; ALP: alkaline phosphatase (**Table S1**).

| **Variables** | **Results** |
| --- | --- |
| Age (Year, Mean ± SD.) | 63.808 ± 9.261 |
| Gender (Female/Male) | 15 (57.7%)/11 (42.3%) |
|  | 7 PrPD (26.9%) |
| Operation | 6 PPPD (23.1%) |
|  | 12 Distal pancreatic resections (46.2%) |
|  | 1 Total pancreatectomy (3.8%) |
|  | 13 Head (50%) |
| Location | 5 Corpus (19.2%) |
|  | 8 Tail (30.8%) |
|  | 7 G2 (26.9%) |
| TNM Classiﬁcation Grading | 17 G3 (65.4%) |
|  | 2 G4 (7.7%) |
| TNM Classiﬁcation Tumor | 2 T2 (7.7%) |
|  | 23 T3 (88.5%) |
|  | 1 T4 (3.8%) |
|  | 11 N0 (42.3%) |
| TNM Classiﬁcation Lymph Nodes | 14 N1(53.8%) |
|  | 1 N2 (3.8%) |
| TNM Classiﬁcation Metastasis | 6 M0 (23.1%) |
|  | 20 M1 (76.9%) |
|  | 12 R0 (46.2%) |
| TNM Classiﬁcation Resection | 12 R1 (46.2%) |
|  | 2 R2 (7.7%) |
| **Lab values before operation** |  |
| CEA | 6.052±11.459 |
| CA19-9 | 1258.553±2178.596 |
| Bilirubin (mg/dl) (Mean ± SD) | 1.851±3.592 |
| Albumin (g/L) (Mean ± SD.) | 42.785±5.766 |
| Lipase (U/L) (Mean ± SD.) | 80.456±84.852 |
| Amylase (U/L) (Mean ± SD.) | 72.861±38.791 |
| INR (Mean ± SD.) | 11.45±25.198 |
| Creatinine (mg/dl) (Mean ± SD) | 0.872±0.154 |
| CRP (mg/L) (Mean ± SD) | 1.737±4.457 |
| ALP (U/L) (Mean ± SD.) | 162.916±218.021 |
| GGT (U/L) (Mean ± SD.) | 280.76±744.902 |
| Leukocytes (/mm^3^) (Mean ± SD.) | 7384.231±2793.957 |
| Platelets (/mm^3^）(Mean ± SD.) | 240.923±59.574 |
| Hb (g/dl) (Mean ± SD) | 12.684±2.757 |
| **Primary tumors of PDAC** |  |
| CD3 (concentration) | 157-698.667(median 442.667)/0.145 mm^2^ |
| CD8 (concentration) | 79.667-552.667 (median 362.833)/0.145 mm^2^ |
| CD20 (concentration) | 19-1227 (median 676.5)/0.145 mm^2^ |
| CD66 (concentration) | 0-238 (median 45.167)/0.145 mm^2^ |
| GLUT1 (concentration)  HIF-1α (concentration) | 60.667-450(median 141.333)/0.145 mm^2^  10-205.333(median 93.167)/0.145 mm^2^ |
| PDHK1 (concentration) | 0-170.333(median 46.333)/0.145 mm^2^ |
| **Metastatic tumors of PDAC** |  |
| CD3 (concentration) | 71–780.667 (median 362.833)/0.145 mm^2^ |
| CD8 (concentration) | 0–298 (median 101.833)/0.145 mm^2^ |
| CD20 (concentration) | 0–629.333 (median 155.665)/0.145 mm^2^ |
| CD66 (concentration) | 0–233.333 (median 71.665)/0.145 mm^2^ |
| GLUT1 (concentration) | 16.333-529.333(median 175.167)/0.145 mm^2^ |
| HIF-1α (concentration) | 0-221(median 103.417)/0.145 mm^2^ |
| PDHK1 (concentration) | 0-104.667(median 38.667)/0.145 mm^2^ |

1. Immunohistochemistry staining revealed positive staining of TILs and MCMs (**Figure S1**) in the stromal area of PDAC's primary and metastatic tumors.


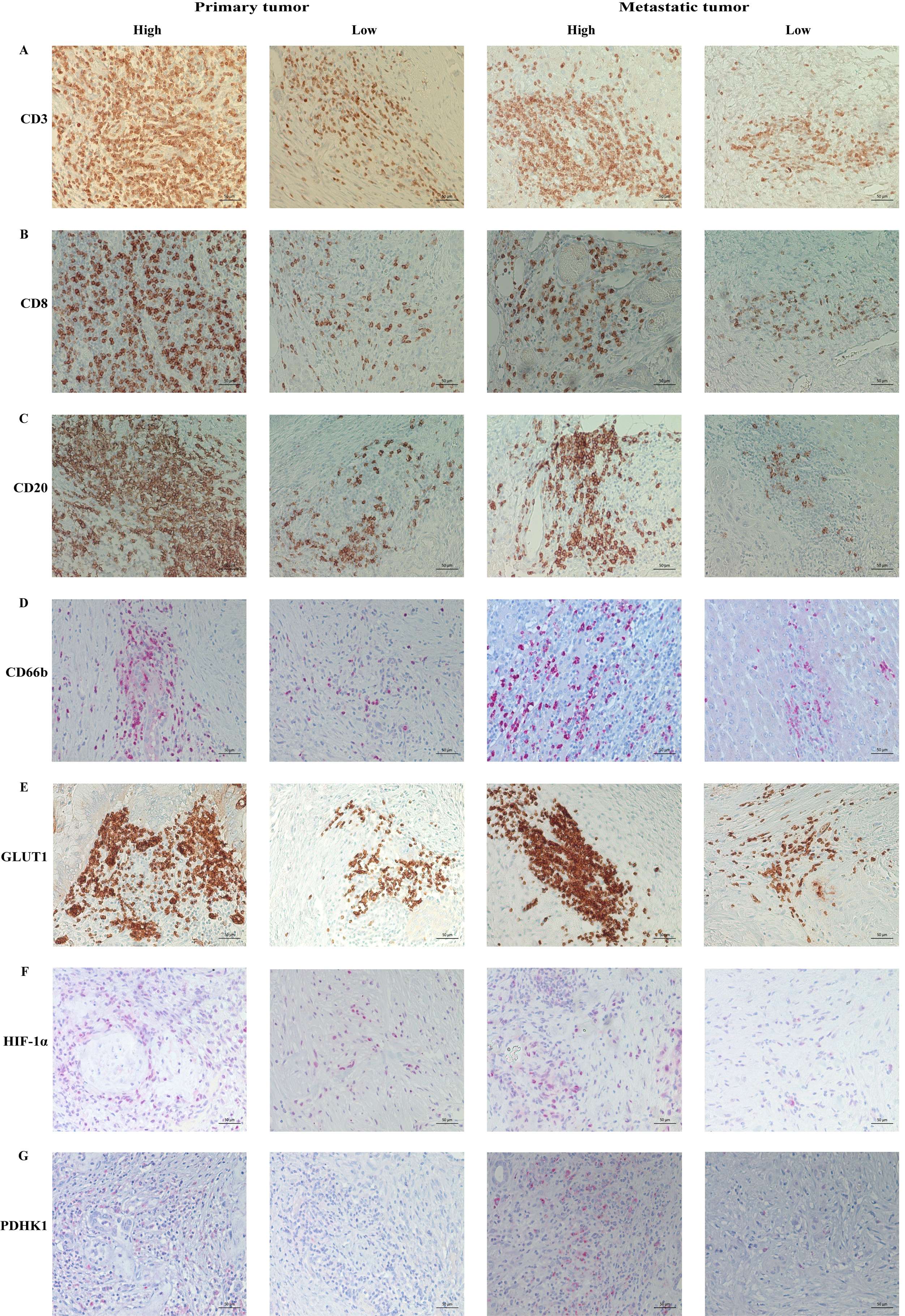


**Figure S1**. Representative pictures of each TILs and MCMs subset by immunohistochemistry staining (* 200). Hot spots with tumor-infiltrating leukocytes of CD3^+^ (A), CD8^+^ (B), CD20^+^ (C), and CD66b^+^ (D) cells, and with tumor-infiltrating metabolic checkpoint molecules of GLUT1 (E), HIF-1α (F), and PDHK1 (G) under a power field of 200x magnification. CD3 is the marker of T cells, CD8—of cytotoxic T cells, CD20—of B cells, CD66b—of neutrophils, GLUT1—of glucose transporter 1, HIF-1α—of hypoxia inducible factor 1α, and PHDK1—of pyruvate dehydrogenase kinase1. Scale bar shows 50μm.

1. TILs in both PDAC primary and metastatic tumors did not show the significance influence on DFS. Kaplan-Meier plots revealed that there were no prognostic values of TILs in both PDAC primary tumors (CD3+ p=0.931, CD8+ p=0.528, CD20+ P=0.458, CD66b+ p=0.312) and metastatic tumors (CD3^+^ p=0.357, CD8^+^ p=0.531, CD20^+^ P=0.076, CD66b^+^ p=0.561) on DFS (**Figure S2**).

**Figure S2**. Survival graphs of disease-free survival for high (blue) vs. low (red) infiltration of CD3^+^, CD8^+^, CD20^+^, and CD66b^+^ in primary tumors(A-D) and in metastatic tumors(E-H). TILs show no prognostic value for DFS.

1. As described in the text. Hot spots of MCMs were observed in the tumor stromal area under 200x

magnification, followed by quantification of the tumor immune stroma (QTiS) to quantify tumor-infiltrating metabolic checkpoint molecules (GLUT1, HIF-1α, and PDHK1).

Next, the quantitative results of GLUT1, HIF-1α, and PDHK1 from the primary and metastatic tumors of 26 metastatic PDAC cases were divided into primary and metastatic groups for pairwise comparison. We found that there was no statistical significance in the difference of MCMs (GLUT1 p=0.44, HIF-1α p=0.72, PDHK1 p=0.46) between the primary and metastatic tumors. **(Figure S3)**

**Figure S3.** Represent the difference of infiltration of GLUT1 (**A**), HIF-1α (**B**), PDHK1 (**C**) in PDAC primary and metastatic tumors, with no statistical significance.

1. MCMs in both PDAC primary and metastatic tumors did not show the significance influence on

DFS. Kaplan-Meier plots revealed that there were no prognostic values of MCMs in both PDAC primary tumors (GLUT1 p=0.085, HIF-1α p=0.148, PDHK1 P=0.069) and metastatic tumors (GLUT1 p=0.537, HIF-1α p=0.788, PDHK1 P=0.244) on DFS (**Figure S4**).

**Figure S4.** Survival graphs of disease-free survival for high (blue) vs. low (red) infiltration of GLUT1, HIF-1α, and PDHK1 in primary tumors (A-C) and in metastatic tumors (D-F). MCMs show no prognostic value for DFS.
